# Neurodegeneration in Autoimmune Optic Neuritis Is Associated with Altered APP Cleavage in Neurons and Up-Regulation of p53

**DOI:** 10.1371/journal.pone.0138852

**Published:** 2015-10-01

**Authors:** Sabine Herold, Prateek Kumar, Sven P. Wichert, Benedikt Kretzschmar, Mathias Bähr, Moritz J. Rossner, Katharina Hein

**Affiliations:** 1 Department of Neurology, University Medicine Göttingen, Göttingen, Germany; 2 Molecular and Behavioral Neurobiology, Department of Psychiatry, Ludwig Maximilians University Munich, Munich, Germany; NIH/NEI, UNITED STATES

## Abstract

Multiple Sclerosis (MS) is a chronic autoimmune inflammatory disease of the central nervous system (CNS). Histopathological and radiological analysis revealed that neurodegeneration occurs early in the disease course. However, the pathological mechanisms involved in neurodegeneration are poorly understood. Myelin oligodendrocyte glycoprotein (MOG)-induced experimental autoimmune encephalomyelitis (EAE) in Brown Norway rats (BN-rats) is a well-established animal model, especially of the neurodegenerative aspects of MS. Previous studies in this animal model indicated that loss of retinal ganglion cells (RGCs), the neurons that form the axons of the optic nerve, occurs in the preclinical phase of the disease and is in part independent of overt histopathological changes of the optic nerve. Therefore, the aim of this study was to identify genes which are involved in neuronal cell loss at different disease stages of EAE. Furthermore, genes that are highly specific for autoimmune-driven neurodegeneration were compared to those regulated in RGCs after optic nerve axotomy at corresponding time points. Using laser capture micro dissection we isolated RNA from unfixed RGCs and performed global transcriptome analysis of retinal neurons. In total, we detected 582 genes sequentially expressed in the preclinical phase and 1150 genes in the clinical manifest EAE (*P* < 0.05, fold-induction >1.5). Furthermore, using ingenuity pathway analysis (IPA), we identified amyloid precursor protein (APP) as a potential upstream regulator of changes in gene expression in the preclinical EAE but neither in clinical EAE, nor at any time point after optic nerve transection. Therefore, the gene pathway analysis lead to the hypothesis that altered cleavage of APP in neurons in the preclinical phase of EAE leads to the enhanced production of APP intracellular domain (AICD), which in turn acts as a transcriptional regulator and thereby initiates an apoptotic signaling cascade via up-regulation of the target gene p53.

## Introduction

Multiple Sclerosis (MS) is generally described as a chronic autoimmune demyelinating disease of the central nervous system in which immune attack by T- and B-cells against myelin sheath leads to focal lesions in the CNS and subsequent neuronal damage [1]. Thus, it is now well accepted that MS is not only an inflammatory disease, but also has neurodegenerative features including axonal transection and neural cell death [2,3]. Until now, the molecular pathways that lead to neuronal damage and cell loss in MS are not completely understood. Gene microarray analyses provide the possibility of identifying molecular regulation pattern in pathological conditions and can be applied to very small amounts of tissue samples [4]. However, due to the limited access to tissues from patients in the acute disease phase and the rapid time kinetics of neuronal damage most of the knowledge of the underlying molecular processes is derived from animal studies.

Experimental autoimmune encephalomyelitis (EAE) is a widely used animal model of MS, which reflects many pathophysiological features of the disease [5,6]. In the present study, we used an active EAE-model in Brown Norway (BN) rats based on immunization with recombinant myelin oligodendrocyte glycoprotein (MOG). This animal model mimics several aspects of the human disease, including the contribution of B- and T-cells to the inflammation, demyelination and cell death [7]. In BN MOG-EAE 90% of immunized animals display severe optic neuritis with apoptotic cell death of retinal ganglion cells (RGCs) [8–10]. RGCs undergo apoptosis already in preclinical stages of EAE [11,12] before overt histopathological changes in the optic nerve occur, suggesting that neurodegeneration starts in the cell bodies of RGCs. Thus, making it more likely that gene-expression changes in neurons are involved in neurodegeneration under autoimmune conditions.

In the present study, we aimed to test this hypothesis by isolating RNA of laser capture microdissected RGCs from frozen sections and performing microarray analysis. We quantified gene expression changes in RGCs in the preclinical as well as in the clinically manifest EAE. Moreover, in comparison, we used RGCs after surgical transection of the optic nerve (ONT) as a neurodegenerative non-inflammatory control. As MOG-induced optic neuritis and ONT both affect RGC axons, these different model systems are most suitable for a direct comparison of neurodegenerative mechanisms under autoimmune inflammatory and non-inflammatory conditions. Moreover, previous studies revealed a similar time kinetic of neuronal apoptosis in both disease models revealing that 40% of RGCs die within the first week after intraorbital optic nerve transection and 41% in the first week after MOG-immunization. This gradual cell loss in the retina extends into the second week, whereas in ONT just 20% and in EAE 27% of RGC of whole RGC-population survives the second week after ONT or after MOG-immunization, respectively [10,13,14]. Therefore, we compared EAE-driven gene-sets to regulated gene-sets detected in RGCs after ONT at the corresponding time points. Comparing transcriptional changes in RGCs in ONT and MOG-EAE enabled us to analyze similarities and differences on the level of RGC-intrinsic mechanisms in both neurodegenerative animal models. The aim of this study was to analyze transcriptional changes in EAE and ONT to generate a hypothetical gene-interaction model in MOG-EAE RGCs that might be involved in neurodegeneration under autoimmune inflammatory conditions. Knowledge about destructive processes in neurons in the context of myelin-directed immune reaction could lead to the development of novel therapeutic strategies that interfere with early mechanisms active in neurons under these specific pathophysiological conditions and thereby delays time point for harsh neurological deficits.

## Materials and Methods

### Rats

Female Brown Norway (BN) rats in the age of 8–10 weeks were used for all experiments. Animals obtained from Charles River (Sulzfeld, Germany) were kept under environmentally controlled and pathogen-free conditions. All efforts were made to minimize the animals suffering. All animal experiments were approved by the local authorities for animal research LAVES (Niedersächsisches Landesamt für Verbraucherschutz und Lebensmittelsicherheit), Oldenburg, Germany.

### MOG- and sham-immunization

Animals were anesthetized by inhalation of isofluorane and injected at the base of the tail with 200 μl inoculum containing 50 μg recombinant rat MOG (kindly provided by C. Stadelmann, Department of Neuropathology, University Medicine Göttingen, Germany) in saline emulsified with complete Freund´s adjuvant (Sigma-Aldrich, St. Louis; MO) containing 200 μg heat-inactivated *Mycobacterium tuberculosis* (strain H37 RA, Difico Laboratories, Detroit, MI). Sham-immunized animals received complete Freund´s adjuvant with heat-inactivated *Mycobacterium tuberculosis* without MOG. Animals were scored for clinical signs of EAE and weighted daily. Clinical score was as follows: grade 0 = no symptoms; grade 0.5 = distal paresis of the tail; grade 1.0 = complete tail paralysis; grade 1.5 = paresis of the tail and mild hind leg paresis; grade 2.0 = unilateral severe hind leg paresis; grade 2.5 = bilateral severe hind limb paresis; grade 3.0 = complete bilateral hind limb paralysis; grade 3.5 = complete bilateral hind limb paralysis and paresis of one front limb; grade 4.0 = complete paralysis (tetraplegia), moribund state or death. In this model the disease generally starts with a clinical score of either 0.5 or 1. Due to the early experimental endpoint, grade 1 was the highest clinical score reached in this study.

### Optic nerve axotomy

Transection of the optic nerve (ONT) was performed as described before [15]. Briefly, rats were anesthetized by intraperitoneal injection with a mixture of 10% Ketamin (0.65 ml/kg; Atatorst GmbH & Co., Twistringen, Germany) / 2% Xylazin (0.35 ml/kg; Albrecht, Aulendorf, Germany) and placed in a stereotaxic frame. After a skin incision was made close to the superior orbital rim, the orbita was opened, taking care to leave the supraorbital vein intact. Following subtotal resection of the lacrimal gland, the superior extraocular muscles were spread by means of a small retractor. The ON was exposed by a longitudinal incision of the eye retractor muscle and the perineurium. Transection was performed approximately 2 mm from the posterior eye pole without damaging retinal blood supply. For pain relief, animals received metamizol in drinking water (3ml/L) from 2 days before the axotomy and continued until day 3 of post-surgical procedure.

### Study design

**Fig 1:**
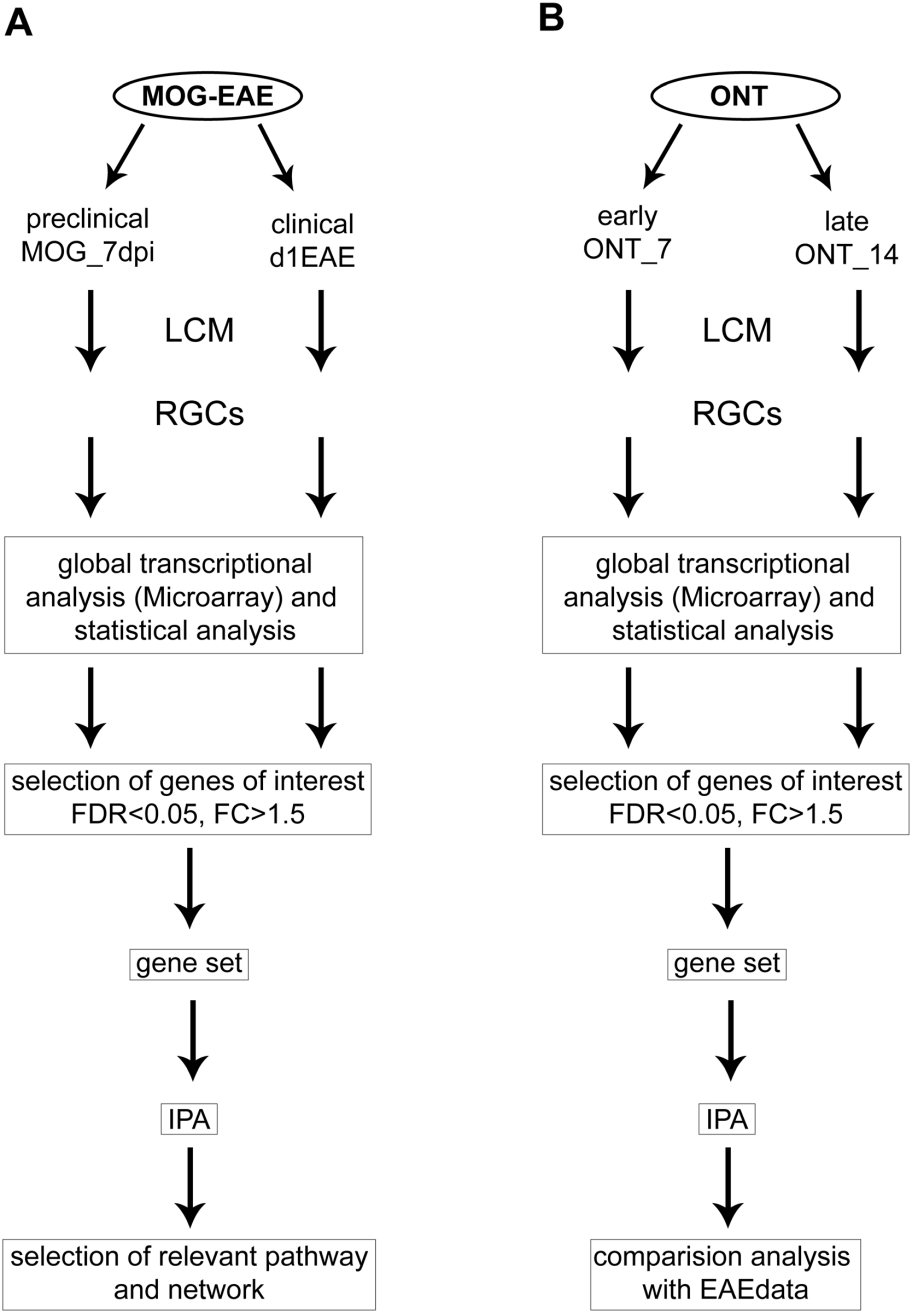
Workflow for data analysis. After sacrificing the animals, eyes were enucleated from the animals immunized with myelin oligodendrocyte glycoprotein (MOG) **(A)** and from the group of animals with optic nerve transection (ONT) **(B)**, at early (day 7 post MOG-immunization (MOG_7dpi) or day 7 after ONT (ONT_7)) and late time points (day 1 of experimental autoimmune encephalomyelitis (d1EAE) or day 14 after ONT (ONT_14)). Retinal ganglion cells (RGCs) were isolated using laser capture microdissection (LCM) and a global transcriptome analysis was performed. Selection of relevant pathways and network specific for MOG-EAE was detected using Ingenuity Pathway Analysis (IPA).

We used 6 different experimental groups of animals which were as follow: 1. MOG-immunized animals analyzed at day 7 post MOG-immunization (MOG_7dpi) or 2. At the day of clinical manifestation of the disease (d1EAE). 3. Sham-immunized control group sacrificed at day 7 post immunization or 4. at day 14 post immunization (Sham). 5. Animals with optic nerve transection (ONT) analyzed at day 7 after axotomy (ONT_7) or 6. At day 14 after axotomy (ONT_14) (Fig 1). 6 eyes per group were used for the microarray and Western blots analysis, 8 eyes were used for quantitative real-time PCR (qPCR) analysis and 3 eyes for immunohistochemistry. Animals were sacrificed using an overdose of carbon dioxide.

### Preparation of eye cryo-sections for laser capture microdissection

Sections mounted on polyethylene naphthalate membrane glass slides (for micro region isolation) were stained prior to dehydration. Slides were fixed in 70% EtOH for 30 sec, rehydrated in H_2_O for 10 sec stained in 2% thionin staining solution (Sigma) washed two times in fresh H_2_O. For dehydration sections were subsequently fixed in 70% EtOH for 30 sec, followed by a dehydration series in 95% EtOH for 30 sec, two times ‘freshly’ pured 100% EtOH for one minute and two times xylene for two minutes. After dehydration slides were air dried, transported and kept in a box with desiccant. Sections for single cell isolation were only subjected to dehydration procedure. We used only precooled RNAse free plastic ware and solutions.

### Laser capture microdissection and microarray

RGC micro regions were micro dissected from 10–15 adjacent slides and were pooled. For the microdissection, we used the Arcturus Veritas microdissection system. For cutting a UV laser (Power = 5–10%) and for attachment an IR laser (Power: 80–90mW and pulse duration: 2500–5000 μs) was used. Successful cutting and collection steps were subsequently validated in bright-field on the quality control slot of the device. Micro dissected samples were lysed in 100 μl of RNA lysis buffer (Qiagen, Hilden, Germany) by vigorous vortexing for 30 sec and stored at -80°C until further use. All procedures were done under RNAse-free conditions. For isolation of minimal amounts of total RNA (100 pg—100 ng) the RNeasy micro kit (Qiagen, Hamburg, Germany) was used according to manufacturer’s instructions. Same modifications as for standard RNA purification were applied. For precipitation of RNA 2 μl of Pelletpaint (Calbiochem, Massachusetts, USA) carrier were added to 100 μl of RNA. After vigorous vortexing for 20 sec, 50 μl of 7.5 M ammonium acetate were added, vigorously vortexed for 20 sec and 360 μl of 100% EtOH was added to the RNA mixture. The RNA solution was centrifuged for 30 min at maximum speed with a micro centrifuge at 4°C. Pellet was washed with 300 μl of 70% ethanol centrifuged at full speed for 10 min, supernatant was discarded and the pellet air dried at RT. 5 μl of RNase-free ddH_2_O was added to the pellet and put on ice for a further 15 min to dissolve.

Quality and concentration of RNA were examined with the Nano labchip and the Bioanalyzer2000 (Agilent Technology, Santa Clara, USA). Quantity and purity was also determined using a Picodrop Spectrophotometer (Picrodrop Limited).

Total RNA of pooled single cells or micro-regions was resuspended with pretested T7-tagged dT21V oligonucleotides. Two-round T7-RNA polymerase-mediated linear amplification was performed according to optimized protocols for low-input RNA amounts (see Small Sample Target Labeling Assay Version II; Affymetrix). Biotin-labeled second-round aRNA was generated with a NTP-mix containing Biotin-11-CTP and Biotin-16-UTP (PerkinElmer, Boston, MA) (2 mMf.c.). Biotin-labeled amplified RNA (aRNA) size distribution and quantity was analyzed with the Agilent 2100 Bioanalyser using the RNA 6000 Nano LabChip kit (Agilent Technologies, Boeblingen, Germany). Samples with lower size compressed RNA products were discarded.

For hybridization at least 5 μg of labeled cRNA was fragmented by heating the sample to 95°C for 35 min in a volume of 20 μl containing 40 mMTris acetate, pH 8.1, 100 mMKOAc, and 30 mM MgOAc. Fragmentation was checked by alkaline agarose electrophoresis. Hybridization, washing, staining, and scanning were performed under standard conditions as described by the manufacturer. Rat Genome 230 2.0 genechips were used that contain over 31,000 probe sets representing transcripts and variants from over 28,000 well-characterized rat genes.

### 3' IVT Expression Analysis

DNA-Microarray data were analyzed with Partek Genomic Suite v6.4 (www.partek.com). Data was pre-processed, which included probe level RMA background correction, quantile normalization across all arrays, and log_2_ transformation followed by median polish to summarize probes to obtain the overall score for each probe set.

The data were filtered based on the expression values of each probe set within the replicate set for each sample; if the expression value of a probe set was below 3.5 (log_2_ value) in all samples, the probe set was removed from the analysis. In order to identify probe sets that were significantly different between samples A and B, a one-way ANOVA statistical test was performed on normalized and filtered probe set level intensities between each group to generate *P*-value and fold change values. Sorting of gene sets by fold change > 1.5 or > -1.5 and a false discovery rate of 5% (*P* < 0.05) was performed with IPA software (Ingenuity Systems, www.ingenuity.com) (Fig 1).

### Quantitative Real time (qRT)-PCR

Retinae were dissected from eye ball and transferred immediately to RNAlater (Ambion, Darmstadt, Germany) and stored at -20°C for further use. Total RNA was isolated by using RNeasy mini kit (Qiagen, Hilden, Germany) according to manufacturer’s instructions. The concentration of RNA was determined with Nanodrop 2000 spectrophotometer (Thermo Scientific, Germany). An absorbance ratio of 260 nm to 280 nm was taken as a purity grade. Integrity of RNA was checked by running denatured RNA gel electrophoresis in RNase free condition by using 1X MOPS buffer with 1 μg of total RNA isolated from each retina and looked for two distinct sharp band from each sample which represents 28S and 18S rRNA bands. First-strand cDNA was synthesized for qPCR by using SuperScript® VILO™ cDNA synthesis kit (Life technology, Carlsbad, USA) from 1 μg of total RNA according to the manufacturer’s instructions. The cDNA was stored at -80°C for further use.

The quantification of mRNA of various genes was measured by q-PCR using CFX96™ Real-time System (BioRad, Munich, Germany) and the Fast SYBR® Green Master Mix (Applied Biosystems, Darmstadt, Germany). Calculations of gene expression were performed using the ΔΔCt method according to Pfaffl and colleagues [16]. Ribosomal protein L13 (Rpl-13) served as reference genes for this assay. Primer-sequences for each gene of interest are shown in S1 Table.

### Western blot

For Western blot analysis, retinae were dissected and homogenized in RIPA lysis buffer (cell signaling; cat no: #9806; Germany) supplemented with phenylmethylsulfonyl fluoride (PMSF) (cell signaling; cat no: #8553; Germany) according to the manufacturer's instructions. After centrifugation at 13,000 rpm for 10 minutes cell debris were removed, and protein concentration of the supernatant was measured using BCA assay kits (Thermo Fisher Scientific Inc; U.S.A). Equal amount of protein from each sample was subjected to 12% SDS-PAGE or to 10–20% Mini PROTEAN^®^ Tris-Tricine-PAGE (BioRad; cat no: 456–3113, Munich, Germany) for beta Amyloid (Aβ) and transferred onto a nitrocellulose membrane. Synthetic peptide Aβ 1–40 (Peptide Speciality Laboratories GmbH, Heidelberg, Germany) was used as a positive control. The membranes were blocked with 5% nonfat dry milk in Tris-buffered saline containing 0.1% Tween 20 (TBST) for 1 hour at room temperature. Incubation with rabbit polyclonalanti-p53 (1:200, Santa Cruz Biotechnology; cat no: sc-6243; Germany), rabbit polyclonal anti-beta Amyloid (1:2000, Abcam; cat no: ab62658, Cambridge, England), mouse monoclonal anti-Vimentin (1:1000, Sigma-Aldrich Biochemistry GmbH, cat no:V6630; Germany), mouse monoclonal anti-CrystallinαB (1:1000, Abcam, ab-13496, UK), rabbit polyclonal anti-Crystallinγ (1:500, Santa Cruz Biotechnology Inc., sc-22746, Texas, USA) and mouse monoclonal anti-β-Actin (1:10,000; Abcam; cat no: ab6276; UK) antibodies were performed overnight at 4°C. After being washed, the membranes were incubated with horseradish peroxidase-conjugated appropriate secondary antibody (1:10000, Jackson Immuno Research Europe; UK) in 5% nonfat dry milk in TBST for 1 hour at room temperature, and immune-reactive proteins were detected using the enhanced chemiluminescence method (ECL; Amersham). Syntetic peptide Aβ 1–40 (Peptide Speciality Laboraories GmbH, Heidelberg, Germany) was used as a positive control. The quantification of each band was performed by densitometric measurement.

### Immunohistochemistry

Histochemical analysis was performed on paraformaldehyde-fixed (PFA 4%), paraffin-embedded 2 μm thick cross sections of eye. Slices were washed in PBS and blocked in PBS containing 10% goat serum and 0.5% of triton X-100. Immunohistochemistry was performed using a rabbit polyclonalanti-p53 (1:100, Santa Cruz Biotechnology; cat no: sc-6243; Germany). As secondary antibody, an anti-rabbit-biotinylated antibody was used (Jackson Immuno Research Europe; UK). Microscopic images of stained eye-section were taken with an Axioplan upright microscope (Zeiss; Germany) using AxioVision 4.8.2 software. Exposure time was kept constant for all investigations of p53-staining in all eye-sections.

## Results

### High overlap of gene sets regulated in the preclinical phase of EAE and ONT_7

Using a 1.5 fold-induction and a false discovery rate of 5%, we detected 582 genes sequentially regulated in the preclinical phase of EAE (MOG_7dpi) and 1053 seven days after optic nerve transection (ONT_7). Furthermore, we detected a sequential regulation of 1150- and 945-genes in the clinical phase of EAE (d1EAE) and at fourteen days after optic nerve transection (ONT_14) respectively.

Because of technical limitations, we were not able to perform quantitative (q) PCR or Western blot validation from same micro dissected tissues we used for microarray analysis. Therefore, we performed qPCR as well as Western blot analysis from whole retinal lysate of animals that were run in parallel with the ones used for microarray analysis. Therefore, all selected proteins for qPCR or Western blot had log_2_-values > 0.5 for basal protein expression and a fold change > 4 and do not necessarily contribute exclusive to neurodegeneration in EAE. Although performed from whole retinal lysates, we were able to validate up-regulation of Vimentin and Crystallin-αB and down-regulation of Crystallin-γC and Crystallin-γD on the RNA and protein level (Table 1).

**Table 1:** Validation of gene regulation. Ribosomal protein L13 (rpl13) and β-actin gene was used as an internal control for quantitative PCR and Western blot analysis respectively. Antibody used for evaluation of crystallin gamma regulation (Cryγ) was not subtype specific.

Gene	Micro array (fold change)	qPCR (fold change)	Western blot (fold change)	Day of sequential expression
Vim	4.94	1.27	1.22	MOG_7dpi
Cryab	16.81	2.04	1.82	MOG_7dpi
Cryγc	-6.59	-2.85	-0.33	d1EAE
Cryγd	-5.81	-1.78	-0.33	d1EAE

To quantify potential similarities or differences in neurodegeneration under auto-inflammatory conditions and after traumatic injury, we separated the gene-sets in up- and down-regulated genes and sorted for genes regulated in the same direction in EAE and ONT at corresponding investigation time points (Table 2).

**Table 2:** Total number of genes regulated in EAE and ONT. In the preclinical phase of EAE (MOG_7dpi) 39.01% of genes overlap with genes found in ONT_7. In clinical phase of EAE (d1EAE) regulated genes do hardly overlap (8.18%) with genes found regulated in ONT_14.

Gene-set	MOG_7dpi	%	d1EAE	%
Overall	582	100	1150	100
Specific for EAE	355	60.99	1056	91.82
Common (same direction)	227	39.01	94	8.18

In the early phase of neurodegeneration we detected a high overlap of regulated genes (39.01%) suggesting similar intracellular mechanisms involved in early neurodegeneration under inflammatory conditions as well as after traumatic injury. In contrast, at the later time point this overlap decreased to 8.18%. We further analyzed the gene sets with IPA (www.ingenuity.com) to investigate differences and similarities between gene-expression in both disease models on the level of predicted molecular pathways.

### APP seems to activate an apoptotic signaling cascades in preclinical EAE that involves p53

IPA-network analysis is based on the connectivity of genes in the datasets, whereby genes are considered to be connected in a direct or indirect way. In contrast to canonical pathway analysis, networks are generated *de novo* based on the input data file. A score based on the ratio between the numbers of genes from the dataset divided by the genes included in the network were generated. With this procedure, we discovered a total of 11 networks (score ≥ 7, focus molecules ≥ 10) (S2 Table) regulated in RGCs in preclinical EAE (MOG_7dpi) and 19 networks (score ≥ 7, focus molecules ≥11) in clinical phase of EAE (d1EAE) (S3 Table). Tables 3 and 4 represent the most significant TOP-5 IPA-networks detected in preclinical and clinical EAE.

**Table 3:** TOP 5 networks detected by IPA in the preclinical phase of EAE (MOG_7dpi).

ID	Top diseases and functions	Score	Focus molecules
1	Neurological disease, nervous system development and function, cell-to-cell signalling and interaction	23	21
2	Connective tissue development and function, embryonic development, organ development	21	18
3	Cell death and survival, cancer, organismal injury and abnormalities	15	16
4	Cell-to-cell signalling and interaction, nervous system development and function, protein synthesis	15	16
5	Neurological disease, psychological disorders, hereditary disorder	13	15

**Table 4:** Networks detected by IPA in the clinical phase of EAE (d1EAE).

ID	Top Diseases and Functions	Score	Focus molecules
1	Cell death and survival, cell morphology, organismal survival	42	35
2	Cancer, organismal injury and abnormalities, reproductive system disease	39	34
3	Cellular compromise, gene expression, cell death and survival	16	20
4	Cell morphology, cellular assembly and organization, neurological disease	14	15
5	Endocrine system development and function, molecular transport, small molecule biochemistry	12	18

Analysis performed by IPA networks revealed that cell death starts already in the preclinical phase of EAE (Table 3, network 3), getting more pronounced in the clinical manifest EAE (Table 4, network 1). Because the goal of the work was to uncover pathways that are specifically active in EAE but not in traumatic injury we performed a comparison-analysis between both pathological conditions. Interestingly, we detected a remarkable difference in potential up-stream regulators of gene-sets. For the preclinical phase of EAE amyloid precursor protein (APP) was identified as a single up-stream regulator, detectable with a z-score of 2.15 and an overlapping p-value of 1.26E-1. In the dataset of clinical EAE or in any time point after optic nerve transection APP was not calculated as a putative upstream regulator of gene expression. Thus, our analysis suggested that APP-driven gene-regulation seems to be specific for preclinical EAE and of minor importance for injury-induced cell damage.

Therefore, we generated a network of APP-dependent genes in the dataset of preclinical EAE (Table 5). Here we observed that many of APP-responsive genes overlay with genes involved in cell “death and survival”-associated networks in preclinical EAE suggesting a role for APP in the induction of early cell death under autoimmune conditions.

**Table 5:** APP-responsive genes in dataset of preclinical EAE.

Genes in dataset	Fold change
Tp53	1.816
Tgfbr2	-1.57
Rhoa	1.647
Prlr	-1.576
Kcnd2	1.868
Galk1	2.285
Cdh2	2.111
Nars2	1.8
Atp1b1	2.24
Canx	2.835
Cplx2	1.833
Cycs	1.716
Ext1	1.846
Gnb1	2.131
Kif2a	2.123
Map2k2	1.939
Pacsin1	1.594
Pin1	1.918
Tuba4a	1.653

**Fig 2:**
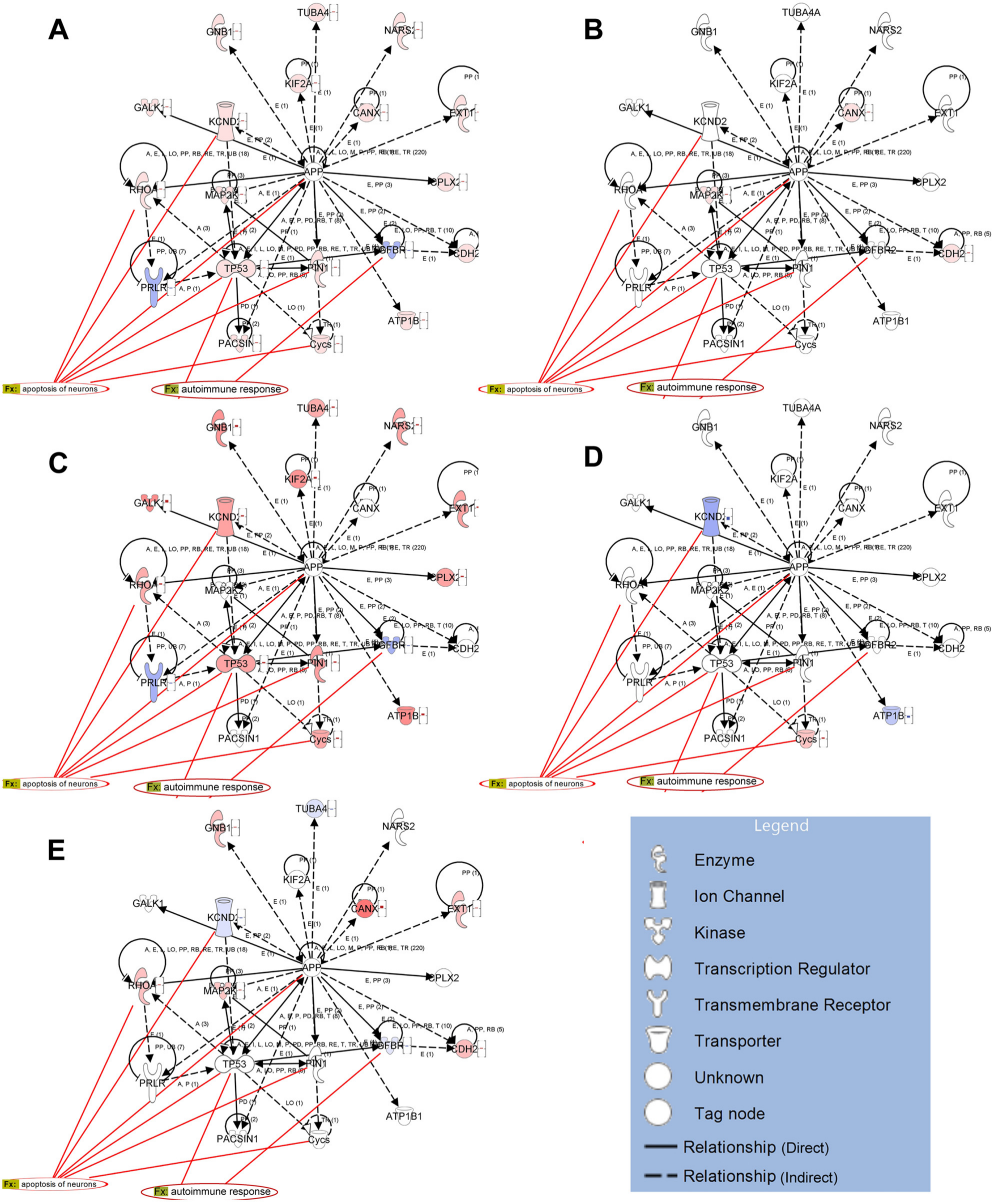
Cell death and survival pathways associated with APP in EAE and optic nerve transection (ONT): **(A)** connection of upstream regulator APP in whole dataset of preclinical EAE (MOG_7dpi), **(B)** set of genes that are commonly regulated in MOG_7dpi and in ONT_7 in the same direction, **(C)** indicate APP-connected genes specific for MOG_7dpi specific genes after subtraction of ONT_7 genes. APP-responsive genes at day of clinical manifestation of the disease (d1EAE) **(D)** and in ONT_14 **(E)**. Pointed arrowheads represent activating relationships, dashed lines indicate indirect activation and solid lines direct activation. Red lines point to genes in the network that are involved in the biological processes “neural apoptosis” or “immune response.

Comparison of APP-responsive gene set in preclinical EAE (MOG_7dpi) with sequentially regulated genes in clinical manifest EAE (d1EAE) as well as/or in ONT revealed that APP-dependent gene-regulation seems to be a specific phenomenon for the early phase of EAE (Fig 2).

**Fig 3:**
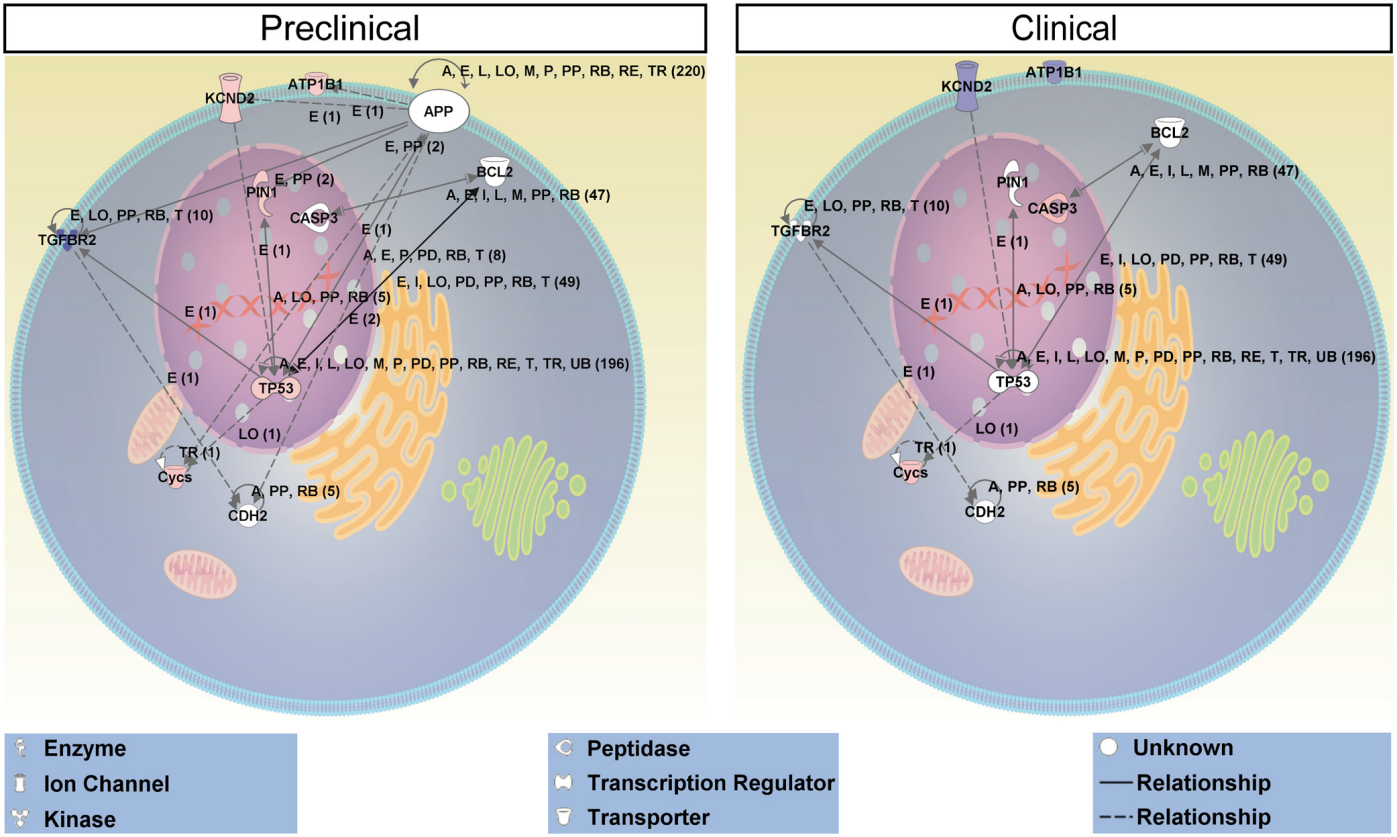
Hypothetical pathway involved in neurodegeneration in preclinical EAE. Red = up-regulation, blue = down-regulation, white = no regulation.

In the dataset of APP-regulated genes we uncovered Kcnd2, Tp53, Pin1, Tgfbr2 and Cycs to be highly involved in “neuronal apoptosis” at early phase of EAE. Furthermore, we found TP53, a key-molecule in cell death and survival pathways to be associated with “autoimmune response” (Fig 2). Based on these findings we generated a hypothetical signalling pathway that might be active in degenerating RGCs in the preclinical phase of EAE (Fig 3). Comparison of this hypothetical pathway with ONT-driven genesets revealed that this neurodegenerative network seems not to be involved in neurodegeneration after traumatic injury (data not shown).

### Validation of hypothetical pathway involved in neurodegeneration in EAE

**Fig 4:**
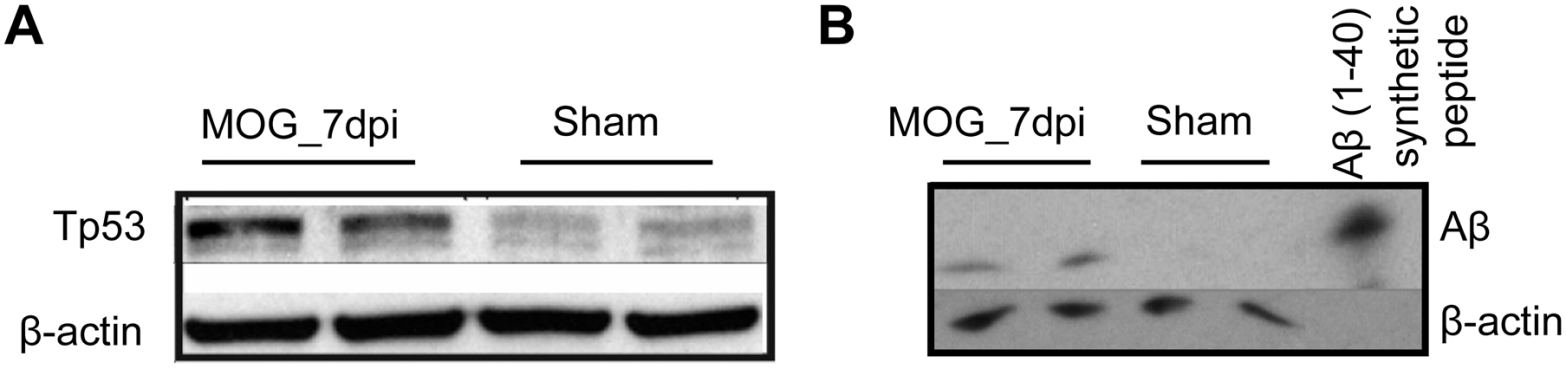
Western blot validation of hypothetical pathway. **(A)** Analysis of protein amount of TP53 in whole retinal lysates of preclinical EAE (MOG_7dpi) and sham-immunized control animals revealed 21% up-regulation of total p53. **(B)** Western blot-analysis of APP-cleavage product Aβ revealed an increase at MOG_7dpi.

To validate the hypothetical pathway generated from microarray data we confirmed up-regulation of the key molecule TP53 (p53) on the protein level by Western blot analysis (Fig 4).

**Fig 5:**
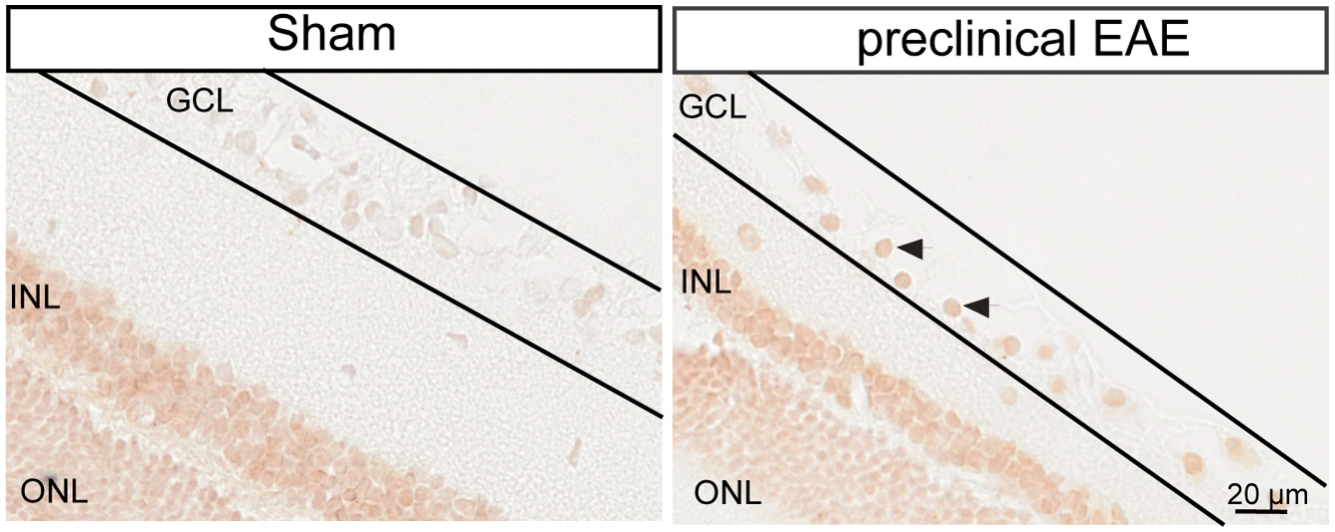
p53-immunoreactivity of RGCs in the preclinical phase of the disease. A strong increase in p53-immunoreactivity was detected in RGC-layer in preclinical EAE (MOG_7dpi). Line indicates borders of the RGC-layer. Arrowheads indicate p53-positive RGCs.

Based on densitometric measurement of band-intensities we detected an increase of 21.5% in p53 in preclinical EAE (MOG_7dpi) compared to sham-immunized control animals. Western blot analysis for full-length APP in retinal lysates suggests that cleavage products of APP seem to be shifted. Furthermore, we performed immunohistochemistry to validate the hypothetical pathway for neurodegeneration in EAE. In healthy control animals we did not detect any p53 positive RGC (Fig 5) whereas in the retina of preclinical EAE an extensive amount of RGCs displayed immune-positive reaction for p53.

## Discussion

The development of potential neuroprotective therapies for MS has to be based on a detailed understanding of molecular and biochemical mechanisms involved in neuronal pathogenesis. Microarray data can provide a mechanistic insight into these pathological processes, especially when interpreted in the context of expression changes that occur in non-auto-immune disease models that we used for the comparison analysis in our current study.

It is well known that complete Freund´s adjuvant (CFA) without an autoantigen induces a complex set of signals to the innate compartment of the peripheral immune system, which in turn may have an effect on the central nervous system (for review see [17–18]). In the present study we aimed to identify pathways exclusively involved in MOG-induced neurodegeneration of retinal neurons and to exclude any effects that may have come from non-antigen specific immune stimulation alone. Therefore, we have used CFA-immunized animals as the control group. In our study, we identified 582 genes sequentially expressed in preclinical EAE and 1150 genes in clinical manifest EAE, suggesting that the immunization with MOG leads to complex gene expression changes in RGCs. Our temporal analysis of gene expression revealed that genes involved in cell death and survival are regulated in an early phase as well as in late phases of EAE. Overall the representation of genes associated with cell death and survival within the dataset is more pronounced in clinical manifest EAE than in the preclinical phase of the disease. Thus, this finding reflects the previous observation that RGC-loss starts in preclinical stages and proceeds in clinical manifest stages of disease. In the preclinical phase of EAE genes like KCNJ5 (G protein-activated inward rectifier potassium channel), Vimentin (Ca^2+^-binding protein) or crystalline-alpha B (heat shock protein) are sequentially expressed suggesting an early dys-regulation of ion-balance and an early stress response within RGCs under autoimmune inflammatory conditions. Our data are thus in accordance with the previous findings obtained from patients with different neurological disease including MS [13,19–21] and glaucoma [22] thereby accounting for a quite widespread role for ion-regulation and up-regulation of the heat-shock protein crystalline-alpha B in neuronal degeneration rather than for a specific phenomenon in neurons under autoimmune inflammatory conditions.

The similarity in neurodegenerative processes in MS and traumatic injury was furthermore proven by the present study, in which we see an overlap of 39.01% of regulated genes in early phases of diseases. In the present study the portion of common genes decreases to 8.19% in later stages of diseases, but still includes genes like caspase 3 and c-Jun, that are involved in apoptotic cell death and are known to be involved in RGC loss under auto-immune inflammation [10,23] as well as in traumatic injury [14]. In summary, the comparison of gene expression profile of RGCs in EAE with traumatic optic nerve transection underline that common pathways in the early phase of the disease are involved in these different diseases with and without the presence of autoimmune inflammation and are in accordance with therapeutic intervention studies successfully targeting different pathways of neurodegeneration in EAE and non-inflammatory lesion (for review see: [24]). However, knowledge of the mechanisms that trigger initial event in neuronal injury in autoimmune inflammation is far from complete.

The main focus of our study was to identify cellular pathways that are specifically involved in the early stage of EAE. To analyze potential differences in neurodegenerative processes in EAE and ONT we performed a direct comparison-analysis with IPA of preclinical EAE- and early ONT-genes. We identified APP as a predicted upstream-regulator of the signaling cascade in the early phase of EAE but not in ONT. APP is an integral membrane protein that is concentrated in neural synapses in the brain under physiological conditions and seems to be involved in synaptogenesis, neuronal survival, neurite outgrowth and neuronal repair [25–26]. Despite this physiological function of APP [27] the protein contributes to neurological diseases like Alzheimer- [28], Huntington- [29] and Parkinson or neuronal injury [30]. From Alzheimer’s disease, it is known that cleavage of APP via the β-secretase leads to the production of neurotoxic Aβ and the APP-intracellular domain (AICD). Previous research from our group revealed accumulation of Aβ in transected neurons [31–32], which strongly supports the idea that cleavage of APP seems to be involved in neural pathology in MOG-EAE. Indeed, we detected changes in the cleavage of APP in preclinical EAE animals compared to sham-immunized controls. Furthermore, we were able to detect a direct target gene, p53 (TP53) of the APP intracellular domain (AICD) in the microarray data set of EAE as well as on the protein level in Western blot analysis and immunohistochemical staining of retinal sections. Thus, our findings support the hypothesis that altered cleavage of APP in RGCs under autoimmune conditions might lead to increase of AICD, which in turn leads to transcriptional up-regulation of p53 [33] and thereby starts a signaling cascade that leads to apoptotic loss of RGCs. That apoptotic cell death is involved in neurodegeneration in EAE and MS was shown in different experimental settings before [10,34–36]. Furthermore, many groups also showed that APP-pathology is implicated in neurodegeneration in MS and EAE [37–40]. Most of the previous work, however, focused rather on the function of toxic Aβ products than regulation of transcription based on AICD signaling in injured cells. In the current investigation, we propose APP as an upstream regulator of EAE-associated genes in preclinical phases of the disease. Based on these findings pharmacological intervention with AICD could thus provide a new avenue for neuroprotective treatment in MS. So far little is known about the impact of APP-dependent gene-regulation via AICD in MS, but pharmacological treatment studies in an animal model of Alzheimer´s disease suggest that disruption of nuclear action of the AICD could improve neurological symptoms [41]. Currently CHF5074 is under clinical intervention for treatment of Alzheimer´s disease. Thus, this substance might not only be of interest to treat conventional neurodegenerative diseases, but might also be tested under autoimmune inflammatory conditions such as MS.

## Conclusions

In the present work, we investigated the mechanisms involved in neurodegeneration under autoimmune inflammatory condition. Here, we observed a high overlap of the genes regulated in auto-immune inflammation and optic nerve transection in the early phase of the disease (day 7 post immunization and day 7 post axotomy). Further we identified APP as a potential upstream regulator only in the early phase of EAE. Furthermore, we also propose that APP initiates apoptotic signaling cascade via up-regulation of p53 in this animal model of MS. Hence, we contribute with this work to the present knowledge about neurodegeneration in MS and may provide new avenues for the development of neuroprotective therapeutics.

## Supporting Information

S1 Table Gene specific primers used in the qPCR. (DOCX)

S2 Table IPA-networks detected in preclinical phase (day 7 post MOG-immunization) of EAE. (DOCX)

S3 Table IPA-networks detected in clinical phase (day 1 of experimental autoimmune encephalomyelitis) of EAE. (DOCX)
