# Supplementary material for: Neurodegeneration in Autoimmune Optic Neuritis Is Associated with Altered APP Cleavage in Neurons and Up-Regulation of p53
Source: PLoS One. 2015 Oct 1;10(10):e0138852. doi: 10.1371/journal.pone.0138852 (PMC4591258; doi:10.1371/journal.pone.0138852)
Supplement: S1 Table — (DOCX) [file pone.0138852.s001.docx]

***Table S1*: Gene specific primers used in the qPCR**

| **Gene** | **Forward (5´-3´)** | **Reverse (3´-5´)** | **Product size (Bp)** |
| --- | --- | --- | --- |
| Vimentin | TCCGCCAGCAGTATGAAAG | CCTCAGAGAGGTCAGCAAAC | 87 |
| Crystallin αB | CTGACCTCTTCTCTACAGCCACT | CGTGCACCTCAATCACGTCTCC | 201 |
| Crystallin γD | GATTACAGAGGCCAGATGGTAG | CAGCACATTGAGGGAGTAGAT | 93 |
| Rpl13 | GGATCCCTCCACCCTATGACA | CTGGTACTTCCACCCGACCTC | 131 |
