# Supplementary material for: Neurodegeneration in Autoimmune Optic Neuritis Is Associated with Altered APP Cleavage in Neurons and Up-Regulation of p53
Source: PLoS One. 2015 Oct 1;10(10):e0138852. doi: 10.1371/journal.pone.0138852 (PMC4591258; doi:10.1371/journal.pone.0138852)
Supplement: S2 Table — (DOCX) [file pone.0138852.s002.docx]

***Table S2*: IPA-networks detected in preclinical phase (day 7 post immunization) of EAE**

| **ID** | **Top diseases and functions** | **Score** | **Focus molecules** |
| --- | --- | --- | --- |
| 1 | Neurological disease, nervous system development and function, cell-to-cell signaling and interaction | 23 | 21 |
| 2 | Connective tissue development and function, embryonic development, organ development | 21 | 18 |
| 3 | Cell death and survival, cancer, organismal injury and abnormalities | 15 | 16 |
| 4 | Cell-to-cell signaling and interaction, nervous system development and function, protein synthesis | 15 | 16 |
| 5 | Neurological disease, psychological disorders, hereditary disorder | 13 | 15 |
| 6 | Nervous system development and function, neurological disease, cell morphology | 13 | 15 |
| 7 | Cell morphology, cellular assembly and organization, cellular development | 12 | 14 |
| 8 | Cell-to-cell signaling and interaction, nervous system development and function, cell death and survival | 11 | 13 |
| 9 | Cell death and survival, cancer, cellular development | 11 | 13 |
| 10 | Behavior, nervous system development and function, cellular assembly and organization | 8 | 11 |
| 11 | Lipid metabolism, small molecule biochemistry, drug metabolism | 7 | 10 |
