# Supplementary material for: Neurodegeneration in Autoimmune Optic Neuritis Is Associated with Altered APP Cleavage in Neurons and Up-Regulation of p53
Source: PLoS One. 2015 Oct 1;10(10):e0138852. doi: 10.1371/journal.pone.0138852 (PMC4591258; doi:10.1371/journal.pone.0138852)
Supplement: S3 Table — (DOCX) [file pone.0138852.s003.docx]

***Table S3***

**IPA-networks detected in clinical phase (day 1 of experimental autoimmune encephalomyelitis) of EAE**

| **ID** | **Top disease and function** | **Score** | **Focus molecules** |
| --- | --- | --- | --- |
| 1 | Cell death and survival, cell morphology, organismal survival | 42 | 35 |
| 2 | Cancer, organismal injury and abnormalities, reproductive system disease | 39 | 34 |
| 3 | Cellular compromise, gene expression, cell death and survival | 16 | 20 |
| 4 | Cell morphology, cellular assembly and organization, neurological disease | 14 | 15 |
| 5 | Endocrine system development and function, molecular transport, small molecule biochemistry | 12 | 18 |
| 6 | Embryonic development, organismal development, nervous system development and function | 12 | 18 |
| 7 | Neurological disease, psychological disorders, metabolic disease | 11 | 17 |
| 8 | Cell-to-cell signaling and interaction, nervous system development and function, behavior | 11 | 17 |
| 9 | Cell morphology, cellular assembly and organization, cellular development | 11 | 15 |
| 10 | Gene expression, embryonic development, organismal development | 11 | 14 |
| 11 | Molecular transport, behavior, nervous system development and function | 10 | 16 |
| 12 | Endocrine system development and function, small molecule biochemistry, lipid metabolism | 9 | 15 |
| 13 | Cell death and survival, liver necrosis/cell death, tumor morphology | 9 | 15 |
| 14 | Lipid metabolism, small molecule biochemistry, molecular transport | 9 | 15 |
| 15 | Cellular assembly and organization, cellular function and maintenance, nervous system development and function | 8 | 13 |
| 16 | Cell death and survival, cellular compromise, liver necrosis/cell death | 8 | 14 |
| 17 | Cellular assembly and organization, nervous system development and function, tissue development | 8 | 14 |
| 18 | Cell morphology, cellular assembly and organization, cellular function and maintenance | 8 | 14 |
| 19 | Embryonic development, nervous system development and function, organismal development | 7 | 11 |
